# Ardipusilloside I induces apoptosis by regulating Bcl-2 family proteins in human mucoepidermoid carcinoma Mc3 cells

**DOI:** 10.1186/1472-6882-13-322

**Published:** 2013-11-21

**Authors:** Xiao-Fang Xu, Tao-Li Zhang, Song Jin, Rong Wang, Xin Xiao, Wei-Dong Zhang, Peng-Yuan Wang, Xiao-Juan Wang

**Affiliations:** 1 Department of Stomatology, 307 Hospital of PLA, Beijing 100071, China; 2 Department of Oral and Maxillofacial Surgery, School of Stomatology, The Fourth Military Medical University, 145 Chang Le Xi Road, Xi’an, Shaanxi Province 710032, China; 3 Department of Pharmaceutical Preparation, School of Stomatology, The Fourth Military Medical University, 145 Chang Le Xi Road, Xi’an, Shaanxi Province 710032, China; 4 Department of Oral and Maxillofacial Surgery, The 22nd hospital of PLA, Middle-Yanqiao Road, Ge’ermu, Qinghai Province 816000, China

**Keywords:** Mucoepidermoid carcinoma, Mc3 cell line, Apoptosis, Bcl-2, Traditional Chinese herb, Ardipusilloside I

## Abstract

**Background:**

*Ardisia pusilla* A. DC., family Myrsinaceae, is a traditional Chinese medicine named Jiu Jie Long with a variety of pharmacological functions including anti-cancer activities. In this study, we purified a natural triterpenoid saponin, ardipusilloside I, from *Ardisia pusilla*, and show that it exhibits inhibitory activities in human mucoepidermoid carcinoma Mc3 cells. We also investigated the underlying mechanisms of proliferation inhibition that ardipusilloside I exerts on Mc3 cells.

**Methods:**

MTT test was used to detect cell proliferation. Cell apoptosis was detected by transmission electron microscopy, Hoechst-33342 staining, DNA fragmentation detection, and flow cytometry. We also used western blot analysis to detect the potential mechanisms of apoptosis.

**Results:**

Ardipusilloside I affected the viability of Mc3 cells in a dose- and time-dependent manner. The IC50 of ardipusilloside I was approximately 9.98 μg/ml at 48 h of treatment. Characteristic morphological changes of apoptosis, including nuclear condensation, boundary aggregation and splitting, and DNA fragmentation, were seen after treatment with 10 μg/ml ardipusilloside I for 48 h. Western blots demonstrated that ardipusilloside I caused Mc3 cell death through the induction of apoptosis by downregulation of Bcl-2 protein levels and upregulation of Bax and caspase-3 protein levels.

**Conclusions:**

Our results revealed that ardipusilloside I could be a new active substance for mucoepidermoid carcinoma treatment. We demonstrated that the potential mechanism of inhibition might be through the induction of apoptosis by regulation of Bcl-2 family protein levels. This suggests a further rationale for the development of ardipusilloside I as an anti-cancer agent.

## Background

Mucoepidermoid carcinoma (MEC) represents one of the most common salivary gland malignancies, accounting for as much as 40–52% of all major and minor salivary gland malignancies [1-3]. To date, chemotherapy and radiotherapy have been largely employed for palliative treatment of MEC metastasis but cancer recurrence and poor prognosis inevitably remain [4]. To explore new active substances or cytotoxic therapies, we have assessed many natural products on MEC cells. In our previous study, we showed that baicalin exhibited anticancer activity in mucoepidermoid carcinoma cell line Mc3 by suppressing cell cycle progression and inducing cell apoptosis [5]. We also found that ardipusilloside I inhibits the proliferation of Mc3 cells in a dose- and time-dependent manner [6].

**Figure 1:**
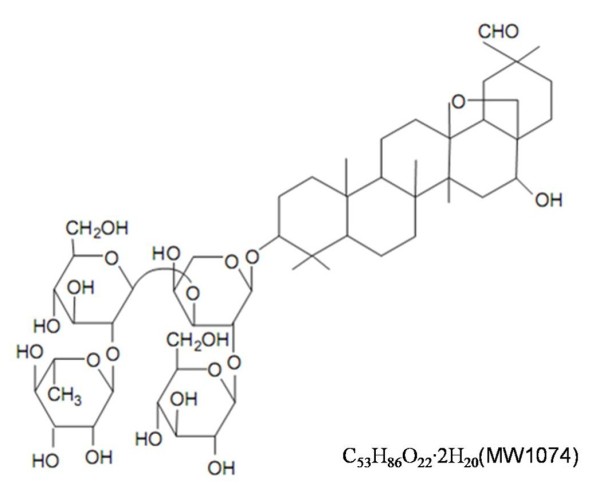
Chemical structure of ardipusilloside I.

Ardipusilloside I (3-*O*-[α-L-rhamnopyranosyl-(1 → 2)-β-D-glucopyranosyl-(1 → 3)-(β-D-glucopyranosyl-(1 → 2))-α-L-arabinopyranosyl]-cyclamiretin A) (Figure 1) is a natural triterpenoid saponin isolated from *Ardisia pusilla* A. DC. (family Myrsinaceae), a traditional Chinese medicine named Jiu Jie Long [7]. Laboratory studies on animals and cell lines have shown that ardipusilloside I inhibits cell proliferation [6], induces cell apoptosis [8,9], and inhibits liver cancer survival, invasion, and metastasis both *in vitro* and *in vivo*[10]. Xiong and colleagues [8] revealed that ardipusilloside I inhibited the growth of human glioblastoma cells, and induced apoptosis through the FasL/Fas signaling pathway. However, little is known about the potential proliferation inhibition mechanism of ardipusilloside I in Mc3 cells.

In the present study, we analyzed the proliferation inhibition of ardipusilloside I on human mucoepidermoid carcinoma Mc3 cells. Our results showed that ardipusilloside I could induce Mc3 cell apoptosis. Moreover, the potential mechanism may be associated with the downregulation of Bcl-2 protein levels and upregulation of Bax and caspase-3 protein levels.

## Methods

### Reagents and chemicals

RPMI 1640 culture medium, M199 medium, and fetal bovine serum (FBS) were purchased from GIBCO BRL (Gibco BRL, Carlsbad, CA). Ardipusilloside I (>98% pure, free of endotoxin) was collected and stored in our laboratory [9], which was dissolved in physiological saline for one night at −4°C, then the supernatant was passed through a 0.22 μm filter (Millipore, Bedford, MA) for sterilization and diluted with culture medium to final concentrations before treatment. In all experiments, the final physiological saline concentration did not exceed 1‰ (v/v), so as not to affect cell growth. Polyclonal rabbit anti -caspase −3 (1:500, Santa Cruz, CA), anti-Bcl-2 (1:500, Santa Cruz, CA), anti-Bax (1:500, Santa Cruz, CA) and anti-β-actin were purchased from Santa Cruz Biotech (CA, USA). 3-(4, 5-dimethylthiazol-2-yl)-2, 5-diphenyltetrazolium bromide (MTT) and Hoechst-33342 were purchased from Sigma(Sigma, USA).

### Cell culture

Human mucoepidermoid carcinoma cell line MEC1 and a highly metastatic human mucoepidermoid carcinoma cell line Mc3 was obtained and stored in our laboratory [11]. MEC1 and Mc3 cells were cultured in RPMI1640 medium supplemented with 10% heat-inactivated (56°C) FBS, 100 U/ml penicillin, 100 μg/ml streptomycin. Human normal parotid acinar cells were obtained by the explant outgrowth technique as reference described [12] from resected parotid tissue of patients with parotid pleomorphic adenoma. The study was approved by the Medical Ethics Committee of the Fourth Military Medical University, and informed consent was signed. Parotid acinar cells were cultured in M199 culture medium supplemented with 20% heat-inactivated (56°C) FBS, 100 U/ml penicillin, 100 μg/ml streptomycin. Cells were incubated at 37°C in a humidified atmosphere containing 5% CO_2_. The cells in exponential phase were used in this study.

### Cell viability assay

The percentage of growth inhibition was determined by MTT assay [13]. In brief, cells were seeded in 96-well microplates at a density of 1 × 10^4^ per well and were cultured for 24 h. After treatment with various concentrations of Ardipusilloside I for 24 h or 48 h, 0.5 mg/ml MTT was added and incubated with cells for 4 h at 37°C in a humidified atmosphere containing 5% CO2. Subsequently, the formazan was dissolved in dimethyl sulphoxide after the medium was removed. Finally the optical density (OD) was measured with an ELX800 reader (Bio-Tek Instruments, Inc., Winooski, VT) at 550 nm. The percentage of cell viability was calculated according to the following formula: (OD value of the control cells –OD value of the treated cells)/ OD value of the control cells × 100%. By definition, the viability of the control cells from the untreated cultures was defined as 100%. The IC 50 value was calculated by SPSS version 16.0.

### S-phase fraction analyses by flow cytometry

The S-phase fraction of Ardipusilloside I treated cells was analyzed by flow cytometry [14]. In brief, 1 × 10^6^ Mc3 cells were treated with 5.0 μg/ml or 10.0 μg/ml Ardipusilloside I for 48 h. All attached cells were collected, washed, suspended in ice-cold PBS, fixed in ice-cold 70% ethanol and stained with 50 μg/ml of PI in the presence of 25 μg/ml of RNase-A. Then cell sorting was performed using FACSCalibur System (Becton Dickinson FACSCalibur, USA) and the histograms were evaluated using CellQuest software. Cellular DNA content was determined by flow cytometric analysis of PI-labeled cells.

### Morphological analysis with fluorescence microscopy

To evaluate the apoptotic activity of Ardipusilloside I, we performed nuclear staining with the DNA-binding dye Hoechst-33342 [15]. In brief, Mc3 cells (1 × 10^6^ cells in 3 ml) were plated into 6-well plates and treated with 10.0 μg/ml of Ardipusilloside I for 24 h or 48 h. Cells were collected by centrifugation at 200 × g for 5 min, washed with ice-cold PBS and then fixed with 2% paraformaldehyde in PBS for 10 min at 4°C. Fixed cells were washed with PBS, incubated with Hoechst-33342 (10 μg/ml) for 15 min in the dark, then placed on slides and observed under a fluorescence microscope (excitation 352 nm, emission 461 nm; NIKON TE2000-E). Apoptotic cells were identified by condensation of chromatin and fragmentation of nuclei. Pictures were obtained using a video camera Q-imaging (Burnaby, BC, Canada).

### Ultrastructural studies by transmission electron microscope (TEM)

Mc3 cells were seeded at the concentration of 1 × 10^5^ cells in culture flask and incubated for 24 h. Cells were then exposed to 10.0 μg/ ml of Ardipusilloside I for 24 h or 48 h. The collected cells were harvested and fixed with 2.5% phosphate-buffered glutaraldehyde at 4°C overnight. The fixed cells were washed with phosphate buffer, post-fixed with PBS containing 1% OsO4, dehydrated in graded alcohol solutions, and embedded. Ultrathin sections (50 nm) were stained with uranyl acetate and lead citrate, and cell morphology was observed by TEM (JEOL, Japan).

### DNA fragmentation detection

The induction of apoptosis by Ardipusilloside I was also tested by the formation of DNA ladder fragments [16]. In brief, Mc3 cells (5 × 10^6^/sample, both attached and detached cells) cultured with Ardipusilloside I (2.5,5.0,10.0 μg/ ml) or without Ardipusilloside I at 37°C for 48 h were harvested and suspended in 1 ml medium. According to the manufacturer’s protocol, the total DNA was isolated by using a DNA extraction kit (TIANGEN BIOTECH, Beijing, China) and electrophoresed on a 1.5% agarose gel containing 0.1 mg/ml ethidium bromide. Ladder formation of oligonucleosomal DNA was photographed under transmitted ultraviolet light.

### Apoptosis analysis by flow cytometry

Apoptosis was measured using flow cytometry to quantify the levels of detectable phosphatidylserine on the outer membrane of apoptotic cells. In brief, Mc3 cells treated with or without Ardipusilloside I for 24 h or 48 h were collected by trypsinization and washed twice with PBS, then fixed in ice-cold 70% (v/v) ethanol at 4°C. After centrifugation, the cell pellets were resuspended in a 500 μl binding buffer solution (10 mM HEPES–NaOH, pH 7.4, 140 mM NaCl, 2.5 mM CaCl2). Then, 5 μl of Annexin V-FITC (BD Pharmingen, USA) and 2 μl of PI solutions were added and the mixtures were further incubated in the dark for 30 min at room temperature. The Annexin V-FITC and PI fluorescence of cultured cells were analyzed by flow cytometry (Becton Dickinson FACSCalibur, USA). For each sample, the fluorescence of 10,000 cells was gated and counted. The percentage of cells in the upper right (late apoptotic cells), upper left (necrotic cells), lower right (early apoptotic cells) and lower left (viable cells) portion of the scatter plot was calculated for comparison.

### Western blot analysis for apoptosis related proteins

The Mc3 cells were treated with various concentration of Ardipusilloside I (0, 2.5, 5.0, 10.0 μg/ml) for 48 h, respectively. Protein extracts of cells were prepared by lysing cells in RIPA buffer (150 mM NaCl, 1% NP-40, 0.5% sodium deoxycholate, 0.1% SDS, 50 mM Tris–HCl, pH 8), 10 mM EDTA and 1 mM PMSF (Sigma, USA) for 30 min at 4°C. Samples were then centrifuged 15 min at 15,000 × g. The protein concentration on the supernatant was determined by BCA protein Assay. For each sample, equal amounts of lysates were loaded on a 9.0% SDS polyacrylamide gel electrophoresis, and transferred to a nitrocellulose membrane (0.22 lm, Pall, NY, USA). Membranes were blocked for 2 h at the RT with blocking buffer (TBS containing 0.05% Tween 20 (Sigma, USA) and 5% non-fat milk (w/v)). Then, the membranes were incubated overnight at 4°C. Primary antibodies (incubated for 1 h at room temperature) were: rabbit polyclonal anti-Bcl-2, anti-caspase-3 and anti-Bax. They were diluted 1:200, except anti-β-actin (1:1000). Thereafter, the membranes were washed thrice for 10 min with TBS buffer containing 0.05% Tween-20, incubated with antirabbit secondary antibodies (1:3000) for 1 h at RT, and then developed by an ECL system according to the manufacturer’s instructions (Amersham, USA).

### Statistical analysis

All data and results were confirmed by at least three independent experiments and were expressed as means ± standard error of the mean (SE). Statistical analysis was performed using students’t test to compare data in different groups. Calculations were carried out using SPSS version 16.0 and P values less than 0.05 were considered statistically significant.

## Results

### Ardipusilloside I inhibited the proliferation of MEC1 cells and Mc3 cells but did not affect the viability of primary cultured parotid acinar cells

**Figure 2:**
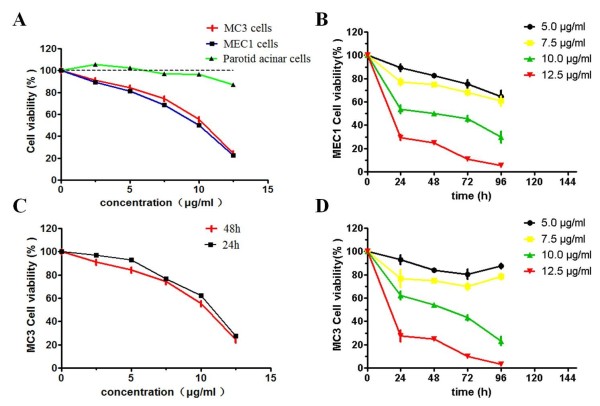
**Effects of ardipusilloside I on the cell viability of human mucoepidermoid carcinoma Mc3 cells. (A)** Exponentially growing of MEC1 cells, Mc3 cells and primary cultured parotid acinar cells were treated with various concentrations of ardipusilloside I (0, 2.5, 5.0,7.5, 10.0, and 12.5 μg/ml) for 48 h. **(B)** MEC1 cells were treated with 5.0, 7.5, 10.0, and 12.5 μg/ml of ardipusilloside I for various times. **(C)** Exponentially growing Mc3 cells were treated with 10.0 μg/ml of ardipusilloside I for 24 h and 48 h. **(D) **Mc3 cells were treated with 5.0, 7.5, 10.0, and 12.5 μg/ml of ardipusilloside I for various times. Cell viability was determined by the MTT assay. Each bar represents the mean ± SE (n = 3).

**Figure 3:**
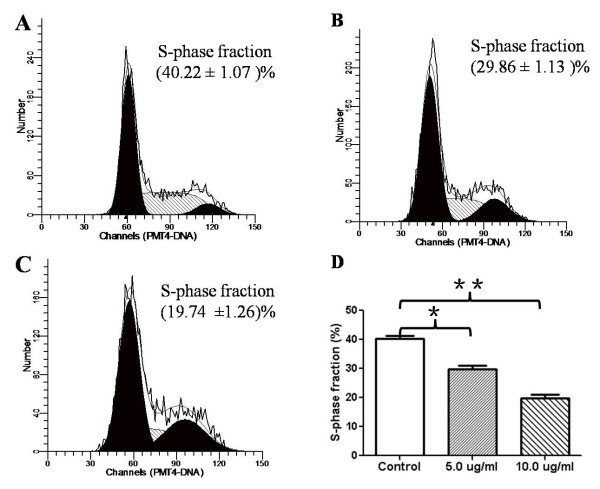
**S-phase fraction in ardipusilloside I-treated and control cells. (A)** Control. **(B)** 5.0 μg/ml ardipusilloside I. **(C)** 10.0 μg/ml ardipusilloside I. **(D)** A significant fraction of cells were found in S-phase when treated with 5.0 μg/ml or 10.0 μg/ml ardipusilloside I for 48 h compared with control cells. *P = 0.0026, **P = 0.0002.

The effect of ardipusilloside I on the viability of Mc3 cell, another mucoepidermoid carcinoma cell line MEC1 cells and primary cultured parotid acinar cells were assessed by the MTT assay. The results showed that low dose of ardipusilloside I did not affect the viability of primary cultured parotid acinar cells, and 12.5 μg/ml of ardipusilloside I for 48 h showed slight cytotoxicity (Figure 2A). The number of viable Mc3 cells and MEC1 cells decreased significantly in a dose- and time-dependent manner compared to vehicle control cells (Figure 2A and B). The viability of Mc3 cells treated with 7.5 μg/ml and 12.5 μg/ml of ardipusilloside I for 48 h decreased to 74.626 ± 0.006% and 24.984 ± 0.095%, and the IC_50_ value was 9.98 μg/ml. (Figure 2C and D). Measurement of the DNA content revealed a significantly lower S-phase fraction in cells treated with 5.0 μg/ml or 10.0 μg/ml ardipusilloside I for 48 h compared with control cells (Figure 3).

### Effects of ardipusilloside I on cell nuclear morphology and DNA fragmentation

To assess whether ardipusilloside I induces apoptosis of Mc3 cells at the single cell level, fluorescence microscopy, electron microscopy, and DNA ladder formation assays were used to detect the cellular nuclear morphology and DNA fragmentation.

**Figure 4:**
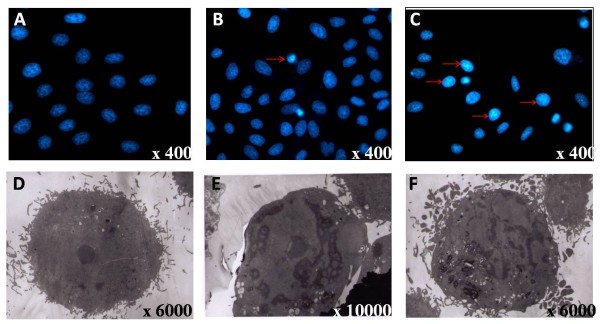
**Chromatin condensation and nuclear fragmentation typical for apoptosis induction were visualized by fluorescence microscopy of Hoechst 33342-stained and TEM.** After treatment with 10 μg/ml of Ardipusilloside I for 24 h **(B)** and 48 h **(C)** or without **(A)** Ardipusilloside I, Mc3 cells were incubated with Hoechst 33342 at a final concentration of 1.5 μM for 10 min. Red arrows show the characteristic morphological changes of apoptosis, including nuclear condensation, boundary aggregation and splitting, and DNA fragmentation. Magnification 400×. **(D)** Mc3 cells in the control group had a normal structure with a large and round nucleus, uniform chromatin density, and clear nucleolus. Magnification 6000×. **(E)** Mc3 cells treated with 10.0 μg/ ml of ardipusilloside I for 24 h showed chromatin condensation. Magnification 6000×. **(F)** Mc3 cells treated with 10.0 μg/ ml of ardipusilloside I for 48 h showed typical cell shrinkage, chromatin condensation, and membrane blebbing. Magnification 6000 ×.

Hoechst 33342 staining of the nuclei revealed that Mc3 cells possessed marked morphological changes in a dose-dependent manner after exposure to ardipusilloside I. Control Mc3 cells had normal morphology with round and homogeneous nuclei (Figure 4A). After treatment with 10 μg/ml of ardipusilloside I for 24 h or 48 h, alterations in the structure, size, and shape of the cell nuclei were detected. Condensed chromatin, cell shrinkage, and nuclear fragmentation as well as the formation of apoptotic bodies were observed (Figure 4B,C). Furthermore, another experiment using electron microscopy clearly showed characteristic apoptotic cells after exposure to 10 μg/ml of ardipusilloside I for 24 h or 48 h. Compared with the normal intracellular morphology of the control cells, after 24 h treatment, condensation of nuclei were observed (Figure 4E). And with 48 h treatment, apoptotic Mc3 cells displayed the distinctive morphological changes of apoptosis, such as condensation of nuclei and the cytoplasm and blebbing of cytoplasmic membranes (Figure 4F).

**Figure 5:**
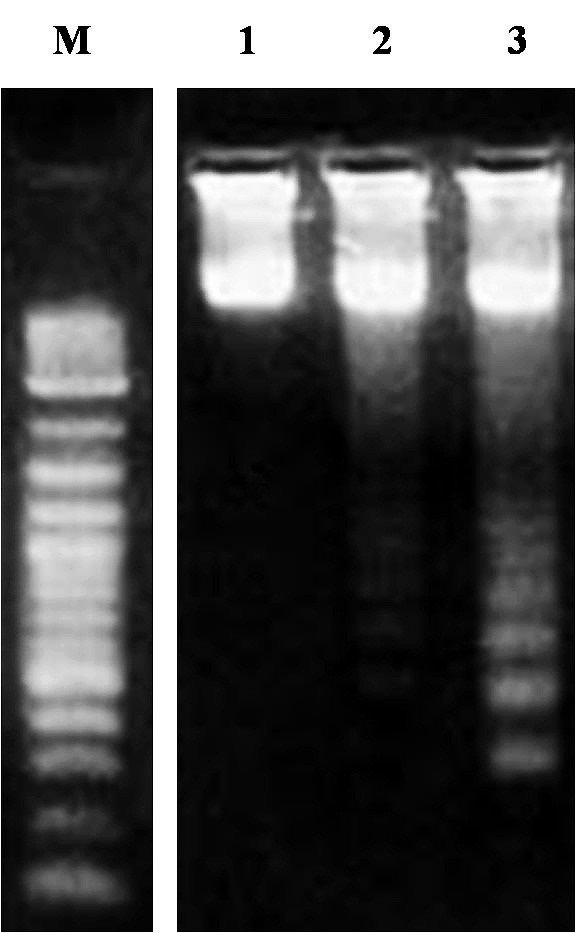
**Nuclear apoptosis induced by ardipusilloside I.** Mc3 cells were cultured with either diluent (control, line 1) or ardipusilloside I (2.5 μg/ml, line 2 and 10 μg/ml, line 3) for 48 h, harvested, and DNA was precipitated and electrophoresed on 1.5% agarose gel. M is the marker.

It is well accepted that DNA ladder formation is highly specific for apoptotic cell death. In Mc3 cells without treatment, no DNA nucleosome ladder was detected (Figure 5, lane 1). In contrast, the typical formation of DNA nucleosome ladders was obvious after treatment with ardipusilloside I, indicating that 10 μg/ml of ardipusilloside induced apoptosis significantly (Figure 5, lane 3).

### Ardipusilloside I induces apoptosis in Mc3 cells

**Figure 6:**
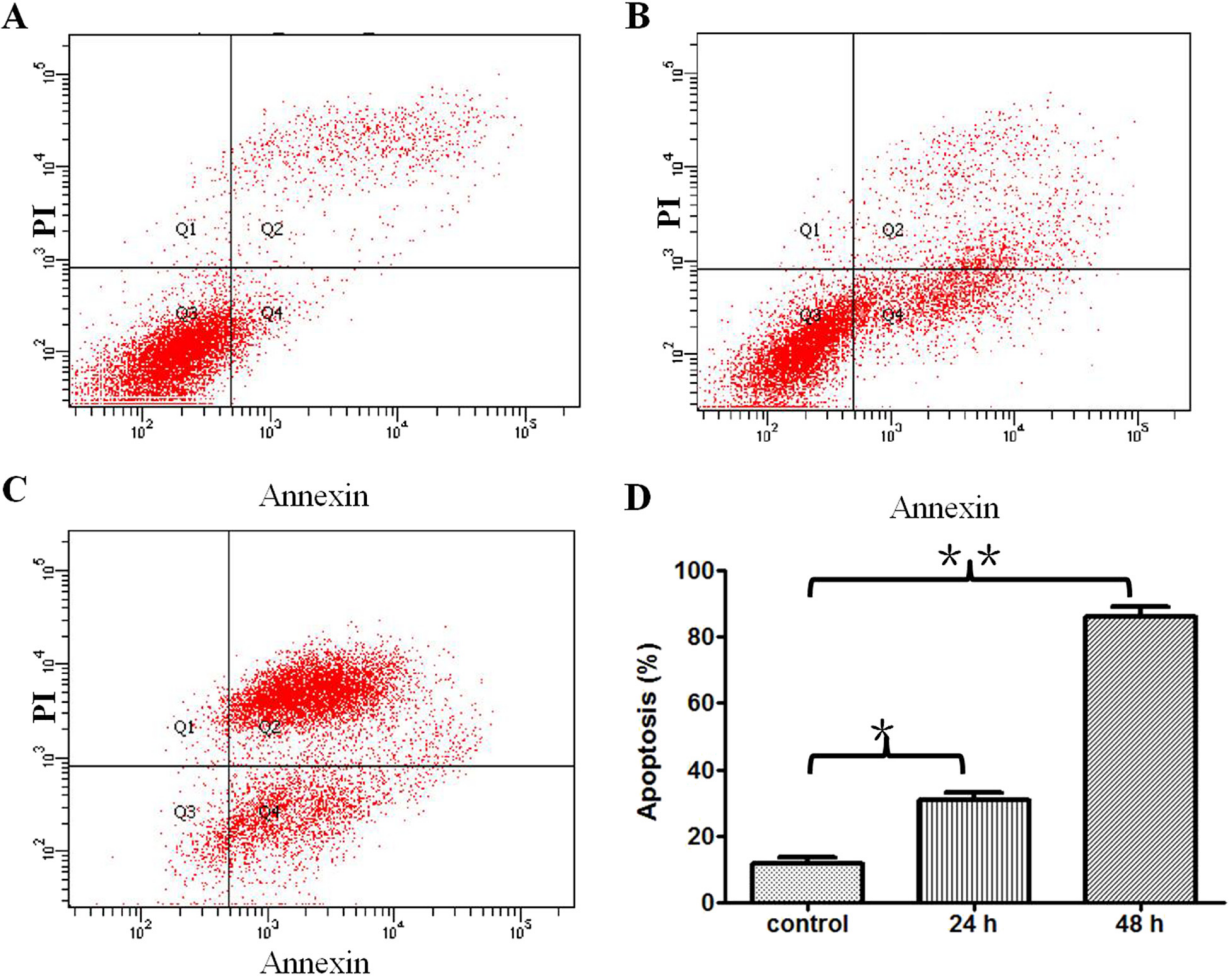
**Ardipusilloside I induces apoptosis of the Mc3 cell line. (A)** Mc3 cells were treated with NS. **(B)** Mc3 cells were treated with 10.0 μg/ml ardipusilloside I for 24 h. **(C)** Mc3 cells were treated with 10.0 μg/ml ardipusilloside I for 48 h. Annexin binding and propidium iodide (PI) staining were analyzed by FACScan. Q3: viable cells; Q4: early apoptotic cells; Q2: late apoptotic cells. **(D**) 10.0 μg/ml ardipusilloside I treatment greatly enhanced the apoptosis of Mc3 cells (*P < 0.0001, **P < 0.0001).

The nuclear morphology of treated cells suggested that ardipusilloside I triggered apoptosis. To confirm that ardipusilloside I induced apoptosis in Mc3 cells, an annexin V binding and PI dual staining assay, which measures another feature of apoptosis, was conducted. We found that 10.0 μg/ml ardipusilloside I treatment for 24 h or 48 h greatly enhanced the apoptosis of Mc3 cells with an apoptotic cell ratio of 86.27 ± 1.86% in the 48 h treated group and 11.63 ± 1.09% in the control group (Figure 6) (*P* < 0.0001).

### Involvement of caspases-3 and Bcl-2/Bax in ardipusilloside I-induced apoptosis

**Figure 7:**
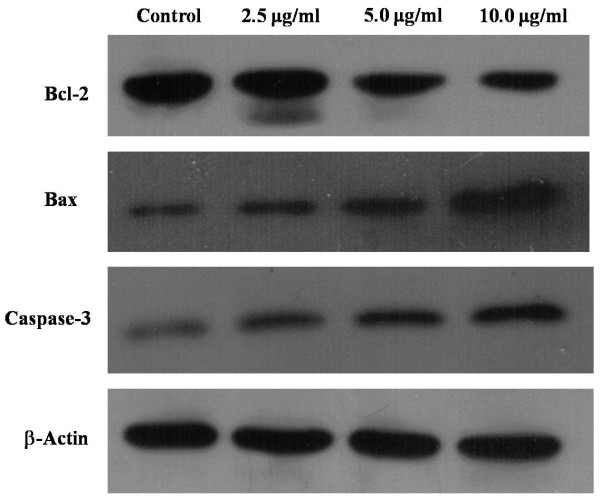
**Effect of ardipusilloside I on the expression of apoptotic proteins.** Mc3 cells were exposed to various concentrations (2.5 μg/ml, 5 μg/ml, and 10 μg/ml) of ardipusilloside I for 48 h and the levels of Bcl-2, Bax, and caspase-3 were measured by Western blot analysis. Increased expression of Bax and caspase-3 and a decrease of Bcl-2 were observed. β-Actin was used as an internal loading control.

To determine the underlying mechanism by which ardipusilloside I induced apoptosis in Mc3 cells, we measured the expression of cell apoptosis molecules caspase-3, Bax, and apoptosis inhibitory protein Bcl-2 (Figure 7). Bcl-2 protein expression was reduced following ardipusilloside I treatment (0, 2.5, 5.0, 10.0 μg/ml for 48 h), while caspase-3 and Bax proteins were increased.

## Discussion

Ardipusilloside I, a natural saponin, has been shown to induce apoptosis in human cervical adenocarcinoma cells, Lewis pulmonary carcinoma, hepatocarcinoma, U87MG cells, and NCI-H460 cells [8,9,17]. Our recent data have also demonstrated that ardipusilloside I significantly decreases the viability of human mucoepidermoid carcinoma Mc3 cells in a dose- and time-dependent manner [6]. In order to explore the potential mechanism, the dose 10.0 μg/ml treatment for 48 h which was closed to its IC_50_ was selected as the main experimental index.

To test whether ardipusilloside I induce apoptosis in human mucoepidermoid carcinoma, cell viability and inhibition were evaluated in Mc3 cells. Morphological studies revealed that Mc3 cells treated with ardipusilloside I displayed obvious apoptotic characteristics such as shrinkage and chromatin margination. Ardipusilloside I induced the apoptosis of Mc3 cells, as detected by annexin V binding and PI dual staining. Apoptosis was confirmed by TEM and nucleosomal DNA ladders, the hallmarks of apoptosis [18]. Our data on Mc3 cells are similar to previous studies in other tumor cell lines in which ardipusilloside I induced tumor cell death with apoptosis [8,9,17]. Therefore, ardipusilloside I may be a tumor cell apoptosis inducer.

As ardipusilloside I-induced apoptosis is well documented through analysis of various cancer cells, recent studies investigated the underlying mechanism of its apoptosis action [8,9,17]. Xiong et al. [8] found that ardipusilloside I enhanced expression of Fas and its ligand FasL in glioblastoma cells. Moreover, they indicated that the ardipusilloside I-induced apoptosis in glioblastoma cells depends on the enhanced expression of the FasL/Fas-signaling pathway and is independent of the activation of caspase-8. However, our result showed that Bax protein expression was increased while Bcl-2 expression was decreased after ardipusilloside I treatment. Meanwhile, caspase-3, the downstream caspase, was overexpressed.

Apoptosis, or programmed cell death, is one of the most important processes regulating the balance of cell growth and cell death, and is preferred to necrosis as a mechanism of cancer cell death [19-21]. Resistance to apoptosis is one of the hallmarks of human cancers. The Bcl-2 family of proteins plays an important role in regulating the mitochondrial or intrinsic apoptotic pathway [22,23]. The apoptotic inhibitory Bcl-2 protein is on the cytoplasmic face of the mitochondrial outer membrane, endoplasmic reticulum, and nuclear envelope, and may participate in mitochondrial permeability transitions [24]. In contrast, Bax is a proapoptotic regulator [25]. Bcl-2 and Bax can be expressed in harmony, and this plays an important role in cell growth and cell death. Cells are active when Bcl-2 is overexpressed while cells die if Bax is overexpressed [26]. It has been reported that overexpression of Bcl-2, which is caused by chromosomal translocation of the Bcl-2 oncogene into the immunoglobulin heavy chain gene locus, is a characteristic feature of human follicular lymphoma [27]. Moreover, the inactivation of the proapoptotic Bax gene resulted by somatic mutations has been identified in certain solid tumors and hematological malignancies. For example, single nucleotide substitutions or frameshift mutations of the Bax gene can occur in mismatch repair-deficient colon cancers or hematopoietic malignancies [28,29].

In this study, we found that ardipusilloside I could markedly reduce the viability of Mc3 cells. We also found that the proapoptotic regulator protein Bax was overexpressed, and anti-apoptotic Bcl-2 protein expression was reduced following ardipusilloside I treatment. Meanwhile, caspase-3, the downstream caspase, was increased. However, there are still some limitations in this present study. First, because it was hard to make primary cultures and subcultures of normal mucoepidermoid acinar cells, the data of ardipusilloside I on these cells are not included in this study. However, Xiong and colleagues tested the effect of ardipusilloside I on human glioblastoma U87MG cells and normal SVGp12 astrocytes [8]. They found that ardipusilloside I did not affect the viability of SVGp12 astrocytes with a dose of 15 μmol/L, while glioblastoma cells were significantly decreased. Second, caspase-3 and PARP are viewed as key enforcers of apoptosis, and activated caspase-3 can further decompose its critical downstream substrate PARP and ultimately lead to apoptosis [30,31]. Unfortunately, we cannot determine this from this data, though the upregulation of Bax and caspase-3 protein levels were studied.

## Conclusion

In conclusion, we demonstrated that ardipusilloside I causes Mc3 cell death through the induction of apoptosis. Its pharmacological mechanism may be associated with the downregulation of Bcl-2 protein levels and upregulation of Bax and caspase-3 protein levels. Our present results provide further rationale for the development of ardipusilloside I as a clinically effective and safe anticancer drug to treat mucoepidermoid carcinoma patients.

## Competing interests

The authors declare that they have no competing interests.

## Authors’ contributions

XXF and ZTL carried out the Cell culture, drug assays, S-phase fraction analyses and drafted the manuscript. WR carried out the Western blot analysis. JS participated in the flow cytometry test and DNA fragmentation detection. XX participated in the Ultrastructural studies by TEM. WPY participated in the design of the study and performed the statistical analysis. WXJ conceived of the study, and participated in its design and coordination and helped to draft the manuscript. All authors read and approved the final manuscript.

## Pre-publication history

The pre-publication history for this paper can be accessed here:

http://www.biomedcentral.com/1472-6882/13/322/prepub
